# Supplementary material for: Complexome profiling of the Chlamydomonas psb28 mutant reveals TEF5 as an early PSII assembly factor
Source: Plant Cell. 2025 Mar 17;37(6):koaf055. doi: 10.1093/plcell/koaf055 (PMC12164590; doi:10.1093/plcell/koaf055)
Supplement: koaf055_Supplementary_Data [file koaf055_supplementary_data.zip › psb28_interactive.zip!/plots/intensity/qualityPlots/peptides/CHLI1_Cre06.g306300.t1.2.html]

### Cre06.g306300.t1.2

Trivial name: CHLI1  
SeqCoverage [median]: 14.40  
Mass: 45.47 kDa  
  
 

| Sequence | MissedCleavage | Mass [Da] | wtIntensity | muIntensity | Score |
| --- | --- | --- | --- | --- | --- |
| AFEPGLLAK | 0 | 944.53 | 637.93 | 854.94 | 91.04 |
| DYEAGQMALTQR | 0 | 1381.63 | 592.34 | 0.00 | 76.23 |
| EGISISHPAR | 0 | 1065.56 | 308.15 | 292.95 | 49.30 |
| IGGVMIMGDR | 0 | 1047.52 | 437.13 | 0.00 | 48.28 |
| STFDENPAAFRK | 1 | 1381.66 | 1198.12 | 678.04 | 78.69 |
| VQIVSQR | 0 | 828.48 | 833.29 | 2657.08 | 111.73 |
|  |  |  | 4007.0 | 4483.0 |  |
